# Real-Time PCR Assay for Rapid and Simultaneous Detection of *vanA* and *vanB* Genes in Clinical Strains

**DOI:** 10.3390/diagnostics11112081

**Published:** 2021-11-10

**Authors:** Hanane Zerrouki, Sid-Ahmed Rebiahi, Linda Hadjadj, Jean-Marc Rolain, Seydina M. Diene

**Affiliations:** 1 Laboratoire de microbiologie appliquée à l’agroalimentaire, au biomédical et à l’environnement, Université de Tlemcen, Tlemcen 13000, Algeria; zerrouki_hanane@hotmail.com (H.Z.); sido8472@yahoo.fr (S.-A.R.); 2 Aix-Marseille Univ., MEPHI, IRD, APHM, IHU-Méditerranée Infection, 13005 Marseille, France; linda.hadjadj@univ-amu.fr (L.H.); jean-marc.rolain@univ-amu.fr (J.-M.R.)

**Keywords:** real-time PCR assay, simultaneous detection, *Enterococci*, *vanA*, *vanB*

## Abstract

Here, we develop a robust and sensitive real-time PCR assay which allows the simultaneous detection of *vanA* and *vanB* genes using common primers. The system was designed using the Primer3 online software. The specificity of primers and probes was first checked by in silico PCR and by BlastN analysis. The genomic DNA of 255 bacterial isolates, including *Enterococcus* spp., Gram-negative, and Gram-positive strains, as well as a collection of 50 stool and 50 rectal swab samples, were tested to evaluate the specificity of the new real-time PCR (RT-PCR) system. The results of the designed RT-PCR were 100% specific and 100% positive on tested vancomycin resistant isolates harboring either the *vanA* or *vanB* gene. RT-PCR assays were negative for all other bacterial species tested including vancomycin-sensitive *Enterococci* and *Enterococcus* strains harboring *vanC* genes. The limit of detection of *vanA* and *vanB* genes by RT-PCR assay was 47 CFU/mL and 32 CFU/mL, respectively. The rapid and accurate detection of vancomycin-resistant Enterococci is the cornerstone for minimizing the risk of nosocomial transmissions and outbreaks. We believe that this assay will strengthen routine diagnostics and surveillance programs.

## 1. Introduction

*Enterococci* strains display both intrinsic and acquired resistance to many antibiotic classes, such as β-lactams, aminoglycosides, fluoroquinolones, and glycopeptides [1]. In the late 1970s, the first vancomycin-resistant *enterococci* (VRE) strains were isolated and, since then, they have spread rapidly worldwide [2], dramatically reducing therapeutic options against *Enterococci* infections [3]. *E. faecalis* and *E. faecium* are the main causative agents for serious nosocomial infections in healthcare settings [4]. Resistance to vancomycin is more frequently encountered in *E. faecium* than *E. faecalis* [5]. The resistance genotypes *vanA* and *vanB* are the most frequently encountered in invasive infections and nosocomial outbreaks [6,7,8]. It is well established that the *vanA* gene confers a high resistance to vancomycin and teicoplanin while the *vanB* gene confers low-level resistance only to vancomycin, which often compromises their detection by phenotypic methods [9]. VRE infections and outbreaks are responsible for antibiotic treatment failures, an increase in morbidity and mortality, prolonged hospital stays, and high healthcare costs [10]. Active surveillance based on rapid and accurate detection of VRE isolates in high-risk patients remains the most effective strategy to reducing all these risks [3]. However, monitoring programs based on phenotypic methods or convention PCR assays are difficult to monitor optimally. However, molecular techniques based on RT-PCR effectively and robustly support these monitoring programs by avoiding the time-consuming and tedious procedures [11]. Herein, we describe for the first time a RT-PCR assay, allowing the simultaneous detection in the same reaction mixture of both *vanA* and *vanB* genes using a common set of primers and two specific probes for *vanA* and *vanB* differentiation.

## 2. Materials and Methods

### 2.1. Design of Primers and Probes

Nucleotide sequences alignment was performed using the ClustalW programme. The specificity of the selected primers and probes was firstly checked using the online “in silico PCR” programme (http://insilico.ehu.es/PCR/ accessed on 30 March 2021) and, secondly, using a BlastN analysis.

### 2.2. DNA Extraction

The sensitivity and specificity of our system were evaluated on the extracted genomic DNA of 255 bacterial isolates, including *Enterococcus* spp., Gram-negative, and Gram-positive strains. One hundred of negative stool (50) and rectal swab (50) samples were also tested. According to the manufacturer’s instructions, DNA was extracted by the EZ1 biorobot (Qiagen, Germantown, MD, USA) with the EZ1 DNA tissues kit from samples and bacterial strains. The stools and rectal swabs were firstly incubated in proteinase K solution at 56°C for four hours. The genomic DNA of bacterial strains was extracted directly from three to five colonies cultured for 24 h on Columbia agar (bioMérieux, Marcy l’Étoile, France).

### 2.3. RT-PCR Assay

The sensitivity of our RT-PCR assay was determined using a series of 10-fold dilutions from an initial inoculum at 10^6^ colony-forming units/mL (CFU/mL) of both *E. faecium* (DSMZ 17050) and *E. faecalis* (DSMZ 12956) reference strains, as positive controls, carrying the *vanA* and *vanB* genes, respectively. The efficiency parameters, slope, and R2 were calculated using a standard curve efficiency established on the basis of the obtained number of log10 CFU/mL and cycle threshold (Ct) values. The limit of detection (LoD) was based on the last dilution detected by an RT-PCR reaction before 35 cycles. To perform the RT-PCR reaction, we used a CFX96 device connected with TM-BioRad using TaqMan technology (Bio-Rad, Lab. Inc., Hercules, CA, USA). The RT-PCR assay conditions were as follows: 50 °C for 2 min, 95 °C for 15 min, and 35 cycles of 95 °C for 1 s and 62 °C for 30 s.

The assay was also tested in an ex-vivo spiking experiment to evaluate their sensitivity and specificity in which negative stool and rectal swab samples were spiked with reference strains carrying *vanA* and *vanB* genes. Five stool samples and five rectal swabs were dissolved in 10 mL of sterile brain heart infusion broth (BHI) (Laboratoire Conda S.A, Torrejón de Ardoz, Spain). Each sample was inoculated using a series of 10-fold dilutions from an initial inoculum at 10^6^ of *E. faecium* (DSMZ 17050) and *E. faecalis* (DSMZ 12956). The DNA extraction and RT-PCR assays were performed from different concentrations, as described above.

## 3. Results

### 3.1. Design of Primers and Probes

**Figure 1:**
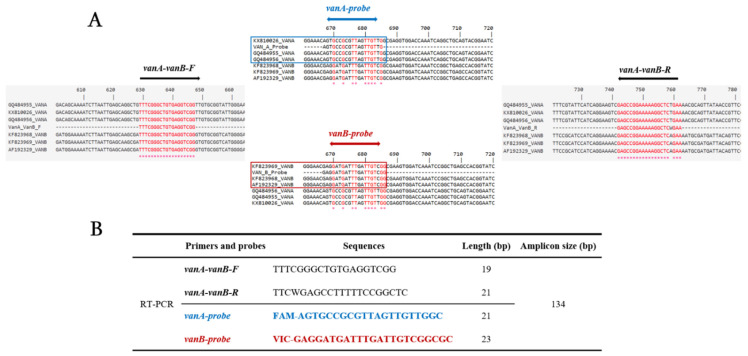
Primer and probe sequences alignment and selected sequence targets. (**A**) sequence alignment of *van* genes for selection of primers and probes; (**B**) Table presenting the selected primers and probes with the size (bp) of each sequence and PCR product.

Nucleotide sequences of *vanA* and *vanB* variant genes were collected from the GenBank database and aligned. As shown in Figure 1, identified sequences discriminated between both genes using different internal bases, which were used to pick the specific probe for each gene. A conserved sequence of 160-bp between *vanA* and *vanB* genes was identified, then conserved, and common bases of both ends were then used as the annealing sites of common primers (Figure 1). The designed primers and probes were checked in silico to avoid misleading amplification of *vanC* gene variants.

### 3.2. Specificity and Sensitivity Tests of the Designed RT-PCR Assay

From extracted genomic DNA of 255 bacterial strains, including 191 *Enterococcus* spp., 26 Gram-negative, 38 Gram-positive strains, and 100 stool and rectal swab specimens, the RT-PCR results were 100% specific and 100% positive on tested vancomycin resistant isolates harboring either *vanA* or *vanB* gene (Table 1). No false-positive was detected by the RT-PCR assay.

**Figure 2:**
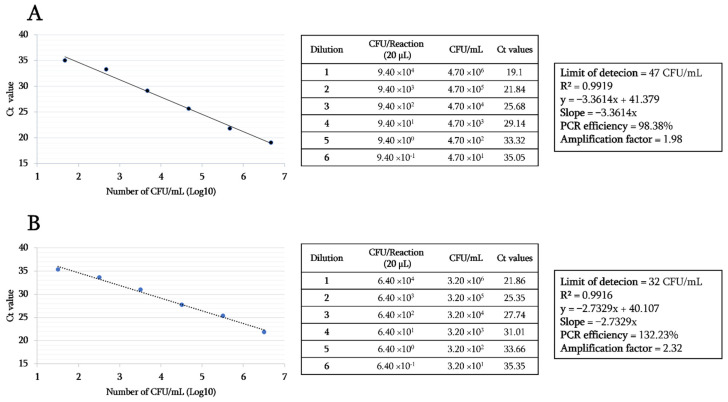
Sensitivity test and limit of detection of *vanA*-*vanB* real-time PCR assay. (**A**) the amplification curves and detection details of the *vanA* gene. (**B**) the amplification curves and detection details of the *vanB* gene.

This RT-PCR was negative for all tested samples and other bacterial species tested, including vancomycin-sensitive *Enterococci* strains harboring the *vanC* gene. The detection limit of the *vanA* gene in *E. faecium* (DSMZ 17050) and the *vanB* gene in *E. faecalis* (DSMZ 12956) was 47 CFU/mL and 32 CFU/mL, respectively. Finally, the results showed the same limit of detection (LOD) value, for both *vanA* and *vanB* genes in samples spiked ex-vivo even in the presence of DNA from other microbes, which are naturally present in stools and rectums. As shown in Figure 2, the dynamic range of the amplification reaction of genomic DNA dilutions of both control strains spanned up to 10^1^ CFU/mL, displaying a correlation coefficient (R²) of 0.9919 for vanA and 0.9916 for vanB genes. The amplification curves were linear and strongly correlated with corresponding Ct values. Replication efficiency was 98.38% and 132.23% with a slope of −3.3614 and −2.7329 for the *vanA* and *vanB* genes, respectively.

## 4. Discussion

Within hospitals, VRE spreads rapidly through patients, caregivers and the environment [12]. Horizontal gene transfer of the *van* operon between *Enterococcus* spp. strains and/or other bacteria can occur frequently. For these reasons, the presence of VREs must be rapidly controlled in health care facilities [4]. Molecular methods have shown to have several advantages compared to phenotypic methods for the detection and distinction of vancomycin-resistance genotypes [11,13]. Moreover, our RT-PCR assay presents a new method that could simultaneously amplify both *vanA* and *vanB* genes in the same mixture reaction. *Clostridium* spp. commonly found in the human gastrointestinal tract that may harbor the different *vanB* variants [14] can induce false-positive reactions in commercial diagnostic kits. It is essential to highlight that, in this study, we tested a large collection of strains, including several bacterial species, including *Clostridium* spp. and also clinical samples (*n* = 100), especially stools and rectal swabs, making the percentage of specificity much more relevant. This system represents a considerable economy in comparison with other RT-PCR systems described previously [9,11,15,16], which required specific primers pair for each gene. The reduced costs and time for diagnostic results are crucial in the management of nosocomial outbreaks. The other molecular support of glycopeptides resistance conferring a low level of resistance to vancomycin, such as the *vanC* gene, is not detected by this system, which could be considered a limitation of this method.

## 5. Conclusions

We believe that our system may be beneficial for hospital settings receiving many critically ill patients and microbiology laboratories in the detection of *Enterococcus* isolates bearing either the *vanA* or *vanB* gene.

## Figures and Tables

**Table 1:** List of the clinical isolates tested for the specificity of the designed RT-PCR assay.

Type of Bacteria	Bacterial Species	Number of Strains	Origin	Probe	
				*vanA*	*vanB*
Gram-positive bacteria	*Staphylococcus aureus*	1	Marseille	−	−
	*Staphylococcus epidermidis*	1	Marseille	−	−
	*Streptococcus agalactiae*	1	Marseille	−	−
	*Streptococcus pneumoniae*	1	Marseille	−	−
	*Staphylococcus haemolyticus*	1	Marseille	−	−
	*Staphylococcus capitis*	1	Marseille	−	−
	*Staphylococcus lugdunensis*	1	Marseille	−	−
	*Streptococcus mitis*	1	Marseille	−	−
	*Staphylococcus warneri*	1	Marseille	−	−
	*Corynebacterium striatum*	1	Marseille	−	−
	*Staphylococcus saprophyticus*	1	Marseille	−	−
	*Corynebacterium jeikeium*	1	Marseille	−	−
	*Staphylococcus simulans*	1	Marseille	−	−
	*Staphylococcus pasteuri*	1	Marseille	−	−
	*Corynebacterium amycolatum*	1	Marseille	−	−
	*Bacillus cereus*	1	Marseille	−	−
	*Staphylococcus cohnii*	1	Marseille	−	−
	*Streptococcus salivarius*	1	Marseille	−	−
	*Streptococcus equinus*	1	Marseille	−	−
	*Corynebacterium propinquum*	1	Marseille	−	−
	*Micrococcus luteus*	1	Marseille	−	−
	*Streptococcus dysgalactiae*	1	Marseille	−	−
	*Staphylococcus hominis*	1	Marseille	−	−
	*C. perfringens*	1	* CSUR (P6929)	−	−
	*C. butyricum*	1	* CSUR (P0102)	−	−
	*P. sordellii*	1	* DSMZ 2141	−	−
	*C. sporogenes*	1	* CSUR (P6393)	−	−
	*C. septicum*	1	* CSUR (P1044)	−	−
	*C. difficile*	5	Marseille	−	−
Total of Strains = 33					
Gram-negative bacteria	*Proteus mirabilis*	1	Marseille	−	−
	*Citrobacter freundii*	1	Marseille	−	−
	*Achromobacter xylosoxidans*	1	Marseille	−	−
	*Enterobacter cloacae*	1	Marseille	−	−
	*Bacteroides fragilis*	1	Marseille	−	−
	*Moraxella catarrhalis*	1	Marseille	−	−
	*Proteus vulgaris*	1	Marseille	−	−
	*Providencia stuartii*	1	Marseille	−	−
	*Haemophilus parainfluenzae*	1	Marseille	−	−
	*Klebsiella pneumonia*	1	Marseille	−	−
	*Pseudomonas aeruginosa*	1	Marseille	−	−
	*Enterobacter kobei*	1	Marseille	−	−
	*Enterobacter asburiae*	1	Marseille	−	−
	*Hafnia alvei*	1	Marseille	−	−
	*Raoultella ornithinolytica*	1	Marseille	−	−
	*Citrobacter braakii*	1	Marseille	−	−
	*Escherichia coli*	1	Marseille	−	−
	*Pasteurella multocida*	1	Marseille	−	−
	*Stenotrophomonas maltophilia*	1	Marseille	−	−
	*Morganella morganii*	1	Marseille	−	−
	*Citrobacter koseri*	1	Marseille	−	−
	*Enterobacter aerogenes*	1	Marseille	−	−
	*Haemophilus influenzae*	1	Marseille	−	−
	*Klebsiella oxytoca*	1	Marseille	−	−
	*Acinetobacter baumannii*	2	Marseille	−	−
Total of Strains = 26					
Vancomycin-resistant *Enterococci* (VRE)	*E. avium (vanA)*	2	Tlemcen	+	−
	*E. casseliflavus (vanA/vanC2)*	1	Tlemcen	+	−
	*E. faecalis (vanA)*	5	Tlemcen	+	−
	*E. faecalis (vanB)*	5	Tlemcen	−	+
	*E. faecium (vanA)*	40	Tlemcen	+	−
	*E. faecium (vanA/vanC1)*	6	Tlemcen	+	−
	*E. gallinarum (* *vanA/vanC1)*	16	Tlemcen	+	−
Vancomycin-susceptible *Enterococci* (VSE)	*E. avium*	2	Tlemcen	−	−
	*E. casseliflavus*	1	Tlemcen	−	−
	*E. casseliflavus (vanC2)*	10	Tlemcen	−	−
	*E. faecalis (vanC1)*	5	Tlemcen	−	−
	*E. faecalis*	50	Tlemcen	−	−
	*E. faecium*	27	Tlemcen	−	−
	*E. otavius*	1	Tlemcen	−	−
	*E. gallinarum*	2	Tlemcen	−	−
	*E. gallinarum (* *vanC1)*	10	Tlemcen	−	−
	*E. hirae*	8	Tlemcen	−	−
Total of Strains = 191					
Reference strains (*van* genes)	*E. faecium (vanA)*	1	DSM 17050	+	−
	*E. faecium (vanA)*	1	DSM 13590	+	−
	*E. faecium (vanA)*	1	DSM 25698	+	−
	*E. faecium (vanA)*	1	DSM 25697	+	−
	*E. faecalis (vanB)*	1	DSM 12956	−	+
Total of Strains = 5					
Human samples	Stools	50	Marseille	−	−
	Rectal sawbs	50	Tlemcen	−	−
Total of Samples = 100					

* DSM: German Collection of Microorganisms and Cell Cultures GmbH. * CSUR: Collection de Souches de l’Unité des Rickettsies.

## Data Availability

Not applicable.
